# African Adders: Partial Characterization of Snake Venoms from Three *Bitis* Species of Medical Importance and Their Neutralization by Experimental Equine Antivenoms

**DOI:** 10.1371/journal.pntd.0003419

**Published:** 2015-02-02

**Authors:** Danielle Paixão-Cavalcante, Alexandre K. Kuniyoshi, Fernanda C. V. Portaro, Wilmar Dias da Silva, Denise V. Tambourgi

**Affiliations:** Immunochemistry Laboratory, Butantan Institute, São Paulo, São Paulo, Brazil; Institut de Recherche pour le Développement, BENIN

## Abstract

**Background:**

An alarming number of fatal accidents involving snakes are annually reported in Africa and most of the victims suffer from permanent local tissue damage and chronic disabilities. Envenomation by snakes belonging to the genus *Bitis*, Viperidae family, are common in Sub-Saharan Africa. The accidents are severe and the victims often have a poor prognosis due to the lack of effective specific therapies. In this study we have biochemically characterized venoms from three different species of *Bitis, i.e., Bitis arietans, Bitis gabonica rhinoceros* and *Bitis nasicornis*, involved in the majority of the human accidents in Africa, and analyzed the *in vitro* neutralizing ability of two experimental antivenoms.

**Methodology/Principal Findings:**

The data indicate that all venoms presented phospholipase, hyaluronidase and fibrinogenolytic activities and cleaved efficiently the FRET substrate Abz-RPPGFSPFRQ-EDDnp and angiotensin I, generating angiotensin 1–7. Gelatinolytic activity was only observed in the venoms of *B. arietans* and *B. nasicornis*. The treatment of the venoms with protease inhibitors indicated that *Bitis* venoms possess metallo and serinoproteases enzymes, which may be involved in the different biological activities here evaluated. Experimental antivenoms produced against *B. arietans* venom or *Bitis g. rhinoceros* plus *B. nasicornis* venoms cross-reacted with the venoms from the three species and blocked, in different degrees, all the enzymatic activities in which they were tested.

**Conclusion:**

These results suggest that the venoms of the three *Bitis* species, involved in accidents with humans in the Sub-Saharan Africa, contain a mixture of various enzymes that may act in the generation and development of some of the clinical manifestations of the envenomations. We also demonstrated that horse antivenoms produced against *B. arietans* or *B. g. rhinoceros* plus *B. nasicornis* venoms can blocked some of the toxic activities of these venoms.

## Author Summary

In this report we have characterized the venoms from three species of *Bitis* snakes involved in accidents with humans in the Sub-Saharan Africa, *i*.*e*., *Bitis arietans*, *Bitis gabonica rhinoceros* and *Bitis nasicornis*. These venoms possess a combination of proteases that can directly affect the coagulation system and the systems involved in the modulation of blood pressure regulation and salt balance *via* generation of vasoactive peptides. We also demonstrated *in vitro* that the deleterious effects of these venoms can be efficiently blocked by experimental horse antivenoms produced against *B*. *arietans* or *B*. *g*. *rhinoceros* plus *B*. *nasicornis* venoms.

## Introduction

In the Sub-Saharan Africa is annually registered approximately 300,000 cases of accidents by snakes which results in 32,000 deaths and a large number of victims with permanent local tissue damage and chronic disabilities [1]. Snakes belonging to the genus *Bitis*, *Viperidae* family, are implicated in many accidents with humans [2]. The genus consist of 16 species, distributed in Africa and Saudi Arabia territories, and presents high intrageneric genetic distance and low monophyly [3]. These snakes differ in size, phenotype and venom composition [4,5]. Molecular data separated the genus *Bitis* in four monophyletic groups. The three West African taxa of the gabonica clade (*Bitis gabonica*, *Bitis rhinoceros*, and *Bitis nasicornis)* were grouped in the subgenera *Macrocerastes*, besides their ambiguous relationship, and *Bitis arietans* was isolated in the subgenera *Bitis* since the bootstrap value does not support any affinity between this species and the others belonging to the genus *Bitis* [3]. Variations were also observed within the same species from different geographic areas complicating the development of effective therapies [5].

The envenomation by *Bitis* often results in severe local damage, hypotension, coagulopathy, thrombocytopenia and spontaneous local bleeding and, in the absence of antivenom therapy, the accident can be fatal [6–8]. *Bitis arietans* is one of the three species of snakes of medical importance in Africa and its venom is considered the most toxic venom of the viper group, based on LD_50_ studies carried on mice [7,9,10]. Besides the severity and high prevalence of the accidents, the biochemical properties of *Bitis* venoms and the mechanism involved in the pathology remain poorly understood. Proteomic and genomic analyses showed that *Bitis* venoms are constituted of proteins belonging to few major families: metalloproteinases, serineproteinases, phospholipases, disintegrins and C-type lectins [4,5,11]. Heretofore, functional studies demonstrated that *Bitis* venom contains metalloproteinases that degrade collagen and fibrinogen [5,12]; a serineproteinase that cleaves kininogen releasing kallidin [13]; lectins that induce calcium release [14]; adenosine that induces mast cell degranulation and hypotension [15]; phospholipases A_2_ (bitanarin) that reversibly blocks muscle-type nicotinic acetylcholine receptors [16]; Arg-Gly-Asp-containing peptides that interfere with platelet aggregation, arietin and gabonin, [17,18]; C-type lectin that binds to the von Willebrand factor interfering with the coagulation cascade, bistiscetin [19], among others.

Therapeutic strategies for treating accidents by snakes belonging to the genus *Bitis*, not always reliable, include antivenom, vasopressors, infusion of platelets and catecholamines [6,8,10]. An in-depth characterization of the venoms belonging to the genus *Bitis* will contribute to a better understanding of the mechanisms by which these venoms cause pathology and shed light on specific therapies targeting the different pathways involved in the envenomation. Thus, the aim of this study was to characterize some toxic properties of the venoms from three species of *Bitis*, *i*.*e*., *Bitis arietans*, *Bitis gabonica rhinoceros* and *Bitis nasicornis*, involved in the majority of the human accidents in Africa, and analyzed the *in vitro* neutralizing ability of two experimental antivenoms.

## Material and Methods

### Reagents

Bovine serum albumin (BSA), gelatin type A, 1,10-phenanthroline (PHE), ethylene diamine tetracetic acid (EDTA), phenylmethylsulfonyl fluoride (PMSF), cetyltrimethylammonium bromide (CTAB), Coomassie Brilliant Blue R-250, Triton X-100, Tween 20, hyaluronic acid, Concanavalin A (Con A) from *Canavalia ensiformis*, Wheat germ agglutinin from *Triticum vulgaris* (WGA), 3, 3”-diaminobenzidine tetrahydrochloride (DAB) and ortho-phenylenediamine (OPD) were purchased from Sigma (Missouri, USA). Goat anti-horse (GAH) IgG labeled with alkaline phosphatase (IgG-AP) or with horseradish peroxidase (IgG-HRPO), 5-bromo-4-chloro-3-indolyl-phosphate (BCIP), nitroblue tetrazolium (NBT) and BCA assay kit were purchased from Promega (Wisconsin, USA). Brij-35 P was purchased from Fluka—BioChemika (Werdenberg, Switzerland). EnzChek Phospholipase A2 Assay Kit was purchased from Invitrogen (California, USA). Fluorescent Resonance Energy Transfer (FRET) substrate, Abz-RPPGFSPFRQ-EDDnp, was synthesized and purified as described [20].

### Venoms

Venoms from *Bitis arietans* (Ba), *Bitis gabonica rhinoceros* (Br; also known as *Bitis rhinoceros*) and *Bitis nasicornis* (Bn) were purchased from Venom Supplies, Tanunda, Australia. These venoms were obtained from males and females snakes, with different ages, captured in Guinea, S. Tome, Angola and Mozambique, and maintained in captivity. Stock solutions were prepared in sterile PBS (10 mM sodium phosphate containing 150 mM NaCl, pH 7.2) at 5 mg/mL based on their protein concentration assessed by BCA assay kit (Promega). Venoms from *Crotalus durissus terrificus* and *Bothrops jaracaca* snakes, supplied by Herpetology Laboratory from Butantan Institute, SP, Brazil, were used as positive controls in the assays for determination of PLA_2_ and hyaluronidase activities, respectively.

### Experimental antivenoms

F(ab’)_2_ fragments generated from antivenoms against *B*. *arietans* (α-Ba) or *B*. *gabonica rhinoceros* plus *B*. *nasicornis* (α-Br+Bn) venoms, as described by Guidolin and collaborators [21], were kindly donated by the Antivenom Production Section from Butantan Institute, São Paulo, Brazil. The neutralization potencies of α-Br+Bn and α-Ba antivenoms were determined as 3.34 mg/mL and 4.62 mg/mL (mg of venom neutralized *per* mL of antivenom), respectively [21]. F(ab’)_2_ fragments from antiserum against botulinic toxin, used as negative control, was kindly donated by Butantan Institute, São Paulo, Brazil.

### Electrophoresis and western blot

Venom samples were separated on 8–16% gradient SDS-PAGE under reducing and non-reducing conditions. The gels were silver stained or transferred onto nitrocellulose membranes. The membranes were blocked with 5% BSA in PBS. Sugar residues were detected with peroxidase labeled Con A or WGA. Reactive proteins were detected using a solution containing 0.1% hydrogen peroxide, 0.5 mg/mL DAB in phosphate buffer (PBS). To determine the specificity and cross-reactivity of Ba and Br + Bn antivenoms against all the three *Bitis* venoms, the nitrocellulose membranes were incubated for 1 h at room temperature (RT) with the antisera diluted 1:1000 in PBS—0.1% BSA. Then, the membranes were incubated with GAH/IgG-AP diluted 1:7500 in PBS, 0.1% BSA, 0.05% Tween for 1 h at RT. Immunoreactive proteins were detected using NBT/BCIP according to the manufacturer’s instructions (Promega).

### Hyaluronidase activity

Hyaluronidase activity was measured as described [22]. Briefly, 30 μg of *Bitis* venoms pre-treated or not with 10 μL of neat Ba or Br + Bn antivenoms were incubated with 25 μL of the hyaluronic acid (0.5 mg/mL) and acetate buffer (0.2 M sodium acetate-acetic acid, pH 6.0, containing 0.15 M NaCl), in a final volume of 100 μL, and incubated for 30 min at 37°C. After incubation, 200 μL of CTAB 2.5% in NaOH 2% was added to the samples. The absorbances were measured at λ 405 nm in a spectrophotometer (Multiskan EX, Labsystems, Finland) against a blank containing hyaluronic acid, acetate buffer and CTAB. All assays were performed in quadruplicate. Results were expressed in units of turbidity reduction (UTR) *per* mg of extract. *Bothrops jararaca* snake venom (30 μg) was used as positive control.

### Phospholipase A_2_ activity

The phospholipase A_2_ activity of B. arietans, B. g. rhinoceros and B. nasicornis venoms were determined using the EnzChek Phospholipase A_2_ Assay Kit (Invitrogen) according to the manufacturer instructions. Briefly, samples of 2.5 μg of the venoms, in a volume of 50 μL of PBS, were mixed with samples of 50 μL of the kit phospholipid FRET substrate, using 96-well microtitre plates, and immediately analysed in a filter-based multi-mode microplate reader (FLUOstar Omega, BMG Labtech, Ortenberg, Germany) at λ_em_ = 515 nm and λ_ex_ = 460 nm and at 37°C. All enzymatic assays were performed in quadruplicate, being the specific activity expressed in UF per minute per microgram of venom. Venom from Crotalus durissus terrificus snake (1 μg) and PBS were used as positive and negative controls, respectively. The venoms of the three Bitis ssp were also submitted to a serum neutralization assay, consisting in a pre-incubation of the venoms with 10 μL of the antivenoms for 30 min at RT, and followed by the addition of the phospholipid FRET substrate and fluorimetic analysis.

### Zymography

Gelatinase activity in the venom samples was analyzed by zymography [23]. Venom samples were pre-incubated (30’ at RT) with 5 mM of the protease inhibitors: ethylenediamine tetraacetic acid (EDTA), 1,10-phenanthroline (PHE) or phenylmethylsulfonyl fluoride (PMSF); or with the antivenoms: α-Ba (10 μL neat) or α-Br+Bn (10 μL neat); or with PBS (positive control), and ran under non-reducing conditions on a 10% polyacrylamide gel containing 1% gelatin type A. The gels were washed twice for 1 h at room temperature in 2.5% Triton X-100, and incubated overnight at 37°C in zymography buffer (50 mM Tris-HCl, 200 mM NaCl, 10 mM CaCl_2_, 0.05% Brij-35; pH 8.3). Gels were stained in Coomassie Brilliant Blue solution (40% methanol, 10% acetic acid, and 0.1% Coomassie Brilliant Blue).

### Cleavage of fibrinogen

Thirty micrograms of fibrinogen were incubated with 1 μg of each *Bitis* spp venoms for 1 h at 37°C with gentle agitation. Venom samples (1 μg) were also pre-incubated (30’ at RT) with EDTA (5 mM), PHE (5 mM), PMSF (5 mM), α-Ba (10 μL neat) or α-Br+ Bn (10 μL neat). After incubation, the samples were separated on 10% SDS-PAGE under reducing condition. The gels were stained with Coomassie Brilliant Blue and the fibrinogenolytic activity determined by the cleavage of alpha, beta and/or gamma chains of the fibrinogen.

### Cleavage of the FRET substrate Abz-RPPGFSPFRQ-EDDnp

*Bitis* venom’s proteolytic activity was determined using a fluorescence resonance energy transfer (FRET) substrate, the peptide Abz-RPPGFSPFRQ-EDDnp. *B*. *arietans* (1 μg), *B*. *g*. *rhinoceros* (2.5 μg) and *B*. *nasicornis* (0.25 μg) venoms were mixed with 5 μM of FRET substrate, in cold phosphate-buffered saline (PBS). The reaction was also performed in the presence of venom pre-treated with PMSF (5 mM), PHE (5 mM), EDTA (100 mM), α-Ba (10 μL) or α-Br+Bn (10 μL) antivenoms. The reactions were monitored by measuring the hydrolysis of FRET substrate in a fluorescence spectrophotometer (Perkin-Elmer, Massachusetts, USA) using 96-well microtitre plates (λ_em_ = 420 nm and λ_ex_ = 320 nm) at 37°C. Control samples were prepared in the presence of an equal volume of buffer or ethanol, used in inhibitors stock solutions. All assays were performed in quadruplicate and the specific proteolytic activity expressed as units of free fluorescence from cleaved substrate *per* μg *per* min (UF/min/μg).

### HPLC analysis of angiotensin I cleavage

Angiotensin I (65 μM) was incubated for 1 h at 37°C with 1 μg of *B*. *arietans* or for 2 h at 37°C with 5 μg of *B*. *nasicornis* or *B*. *g*. *rhinoceros* venoms in phosphate buffer (50 mM sodium phosphate, 20 mM NaCl, pH 7.4). The different time course and venom concentration were used in order to achieve a maximum substrate consumption of 20%. In parallel the venoms were pre-incubated with PHE (5 mM), PMSF (5 mM), EDTA (100 mM), α-Ba (10 μL) or α-Br + Bn (10 μL) antivenoms for 30 min, prior the addition of angiotensin I. Hydrolysis products were analyzed by reverse-phase chromatography (Prominence, Shimadzu) using a Shim-Pack C-18 column (4.6 x 150 mm). The HPLC conditions used for the analytical procedure were 0.1% trifluoroacetic acid (TFA) in water (solvent A), and acetonitrile and solvent A (9:1) as solvent B. The separations were performed at a flow rate of 1 mL/min and a 20–60% gradient of solvent B over 20 min. In all cases, elution was followed by ultraviolet absorption (214 nm) as describe [20]. To determine the scissile bonds in angiotensin I, the fractions were collected manually and submitted to mass spectrometry analysis. The peptide fragments were detected by scanning from *m/z* 100 to *m/z* 1300 using an Esquire 3000 Plus Ion Trap Mass Spectrometer with ESU and esquire CONTROL software (Bruker Daltonics, Massachusetts, USA). Purified 18O-labeled or unlabelled oxidized W derivatives were dissolved in a mixture of 0.01% formic acid: acetonitrile (1:1) and infused in the mass (direct infusion pump) spectrometer at a flow rate of 240 μL/h. The skimmer voltage of the capillary was 40 kV, the dry gas was kept at 5.0 L/min, and the source temperature was maintained at 300°C.

### Antibodies titers and cross-reactivity

ELISA plates were coated with 1 μg/well of *B*. *arietans*, *B*. *g*. *rhinoceros* or *B*. *nasicornis* venoms in PBS (overnight at 4°C). Plates were blocked with PBS-5% BSA and incubated with increasing concentrations of the F(ab’)_2_ antivenoms α-Ba or α-Br + Bn or with the F(ab’)_2_ horse serum against botulin toxin (negative control) for 1 h at 37°C. After incubation, the plates were washed with PBS/0.05% Tween 20 and incubated with a goat anti-horse/IgG-HRPO-conjugate for 1 h at 37°C. Then, plates were washed and the reactions developed with OPD substrate, according to the manufacturers conditions (Sigma). The absorbances were recorded in a spectrophotometer (Labsystems,) at λ 492 nm.

### Statistical analysis

Data were analysed statistically by one way ANOVA followed by Bonferroni multiple comparison test or by two away ANOVA. A *P*-value <0.05 was considered significant.

## Results

### Biochemical analysis of *Bitis* ssp venoms

**Fig 1:**
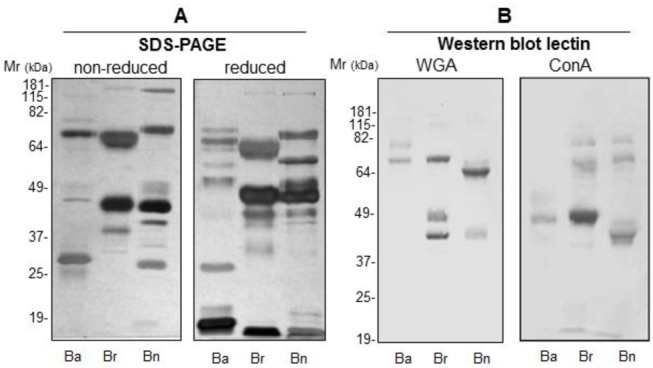
Biochemical characterization of *Bitis* ssp venoms. **[A]** Venoms samples (30 μg) from *B*. *arietans* (Ba), *B*. *g*. *rhinoceros* (Br) and *B*. *nasicornis* (Bn) snakes were separated by SDS-PAGE (8–16% gel) under non-reducing and reducing conditions and silver stained. **[B]** Venom samples (10 μg) from *Bitis* ssp were separated by SDS-PAGE and electrotransfered into nitrocellulose membranes to analyze the presence of oligosaccharide residues. Blots were probed with WGA-HRPO (WGA) or Con A-HRPO (Con A) and the reactions developed with DAB.

All the three *Bitis* venoms presented different electrophoretic profiles containing bands with molecular weight varying from 10 to 200 kDa (Fig. 1A). Some of these bands are probably in complex or present disulfide bonds inter- or intra-chains as observed by the presence of extra bands of lower molecular weight after reduction (Fig. 1A). All venoms presented some proteins with sugar residues, as determined by the interaction with WGA, which selectively binds to N-acetylneuraminic acid and N-acetylglucosamil residues [24] or Con A, which selectively binds to α-mannopyranosyl and α-glucopyranosyl residues [25] (Fig. 1B). The specificity of the lectin binding was confirmed by the absence of bands on the membranes incubated with lectins in the presence of the specific sugars.

### Enzymatic characterization of *Bitis* ssp venoms activities

**Fig 2:**
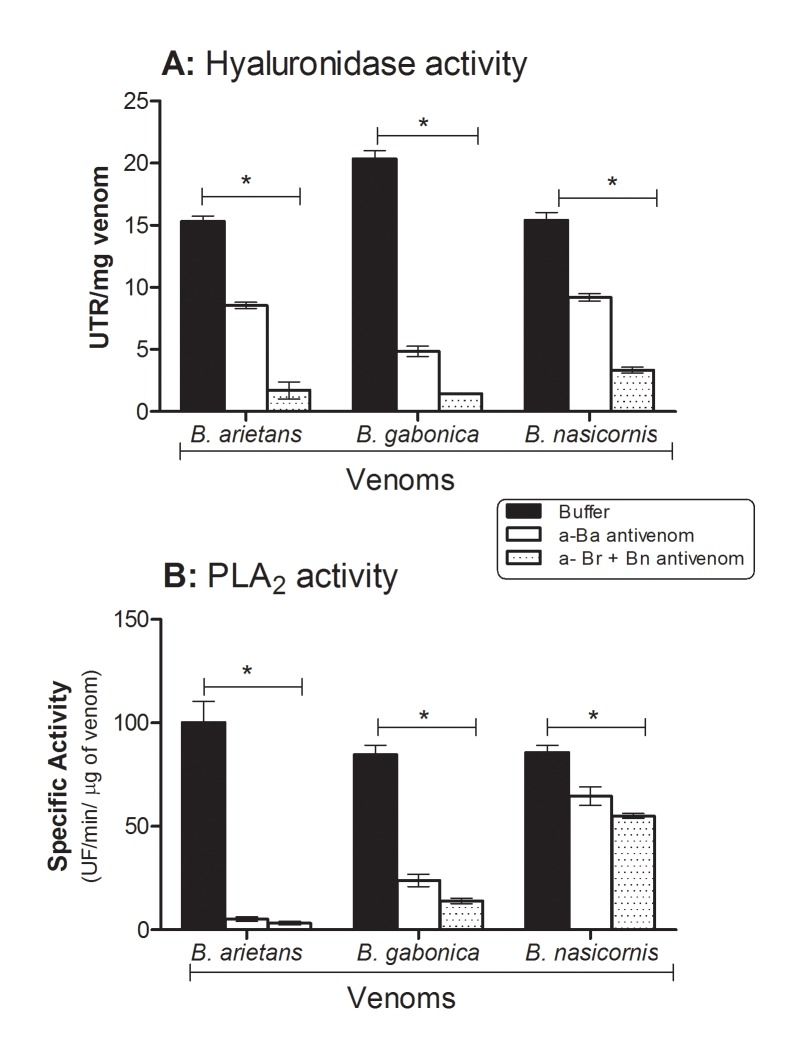
Enzymatic characterization of *Bitis* ssp venoms and the blockage of activities by antivenoms raised against *B*. *arietans* or *B*. *g*. *rhinoceros* plus *B*. *nasicornis*. **[A]** *Hyaluronidase activity*: *B*. *arietans*, *B*. *g*. *rhinoceros* and *B*. *nasicornis* venoms samples (30 μg) pre-incubated with hyaluronic acid at 37°C for 30 min. Alternatively, venom samples (30 μg) were pre-incubated with 10 μL of antivenoms raised against *B*. *arietans* venom (α-Ba) or *B*. *g*. *rhinoceros* plus *B*. *nasicornis* venoms (α-Br + Bn) for 30 min at RT, prior incubation with hyaluronic acid. The turbidity of the mixture was measured in a spectrophotometer at λ_em_ 405 nm. The results are representative of three individual experiments and expressed in units of turbidity reduction (UTR) *per* mg of venom. Statistical analyses were performed using two way Anova analysis (*P< 0.05). **[B]** *Phospholipase A*_*2*_ *activity*: venom samples (2.5 μg) were added to the phospholipid FRET substrate and the fluorescence was immediately measured in a fluorimeter. Alternatively, venom samples (2.5 μg) were pre-incubated with 10 μL of antivenoms raised against *B*. *arietans* venom (α-Ba) or *B*. *g*. *rhinoceros* plus *B*. *nasicornis* venoms (α-Br + Bn) for 30 min at RT, prior incubation with phospholipid FRET substrate. The specific activity was expressed as UF/min/μg. Results are representative of 3 independent experiments performed in quadruplicate. Statistical analyses were performed using two way Anova (*P< 0.05).

Fig. 2A shows that the venoms from *B*. *arietans*, *B*. *g*. *rhinoceros* and *B*. *nasicornis* presented high, but not statistically different levels of hyaluronidase activity. The hyaluronidase activity determined for *Bitis* spp venoms was similar to the one detected in *B*. *jararaca* venom (12 UTR/mg). All *Bitis* venoms, tested in this study, presented similar phospholipase A_2_ activity. The activity of *B*. *arietans* was ~100 UF/min/μg, and of *B*. *g*. *rhinoceros* and *B*. *nasicornis* were ~80 UF/min/μg (Fig. 2B). The phospholipase activity of *C*. *d terrificus* venom, used as positive control of the assay, was 4–5 times higher than the *Bitis* venoms, *i*.*e*., 430 UF/min/μg.

**Fig 3:**
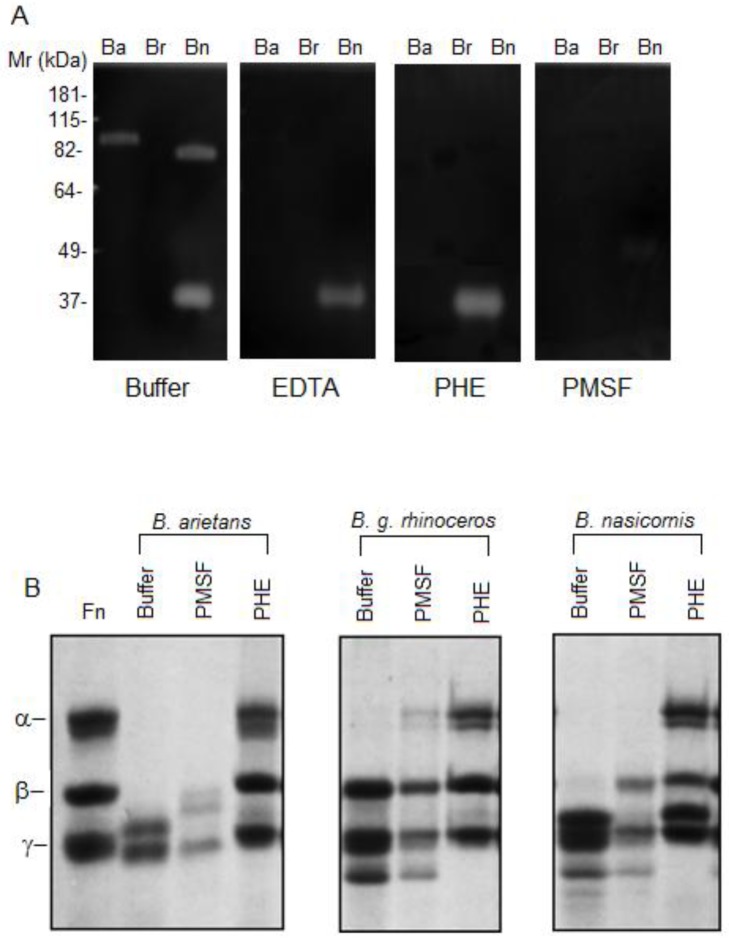
Proteolytic activity of Bitis ssp venoms. **[A]** Gelatinolytic activity analyzed by zymography: Bitis spp venoms (30 μg) were incubated in the absence (buffer) or presence of 5 mM EDTA (EDTA), 5 mM 1,10-phenanthroline (PHE) or 5 mM PMSF (PMSF), submitted to electrophoresis and subsequently incubated overnight at 37°C, in 50 mM Tris-HCl, pH 8.3. Following incubation, the gel was stained with 0.1% Coomassie Brilliant Blue and the gelatinolytic activity was detected as unstained bands on a dark background. **[B]** Fibrinogenolytic activity was assessed using a SDS-PAGE (10%) gel. Thirty micrograms of human purified fibrinogen (Fn) were incubated with 1 μg of Bitis spp venoms pre-incubated or not with inhibitors of serine–(PMSF) or metallo–(PHE) proteinases and ran under reducing condition. The digestion of gelatin and the cleavage of fibrinogen were visualized by Coomassie Brilliant Blue staining.

Zymography analysis showed that *B*. *arietans* and *B*. *nasicornis* venoms have gelatinolytic activity but not *B*. *g*. *rhinoceros* (Fig. 3A). The venom from *B*. *arietans* has a band of ~ 90 kDa with gelatinolytic activity, which was inhibited by EDTA, PHE and PMSF. Moreover, *B*. *nasicornis* venom presented two bands with gelatinolytic activity, ~30 and 80 kDa. The gelatinolytic activity of the band with ~30 kDa was inhibited neither by EDTA nor by PHE and totally inhibited by PMSF.

All venoms cleaved efficiently the fibrinogen’s α chain. In addition, *B*. *arietans* venom also cleaved β and ɣ chains of the fibrinogen while *B*. *nasicornis* also cleaved β chain (Fig. 3B). The fibrinogenolytic activity was strongly inhibited by PHE, demonstrating the involvement of metalloproteinases in this process (Fig. 3B).

**Fig 4:**
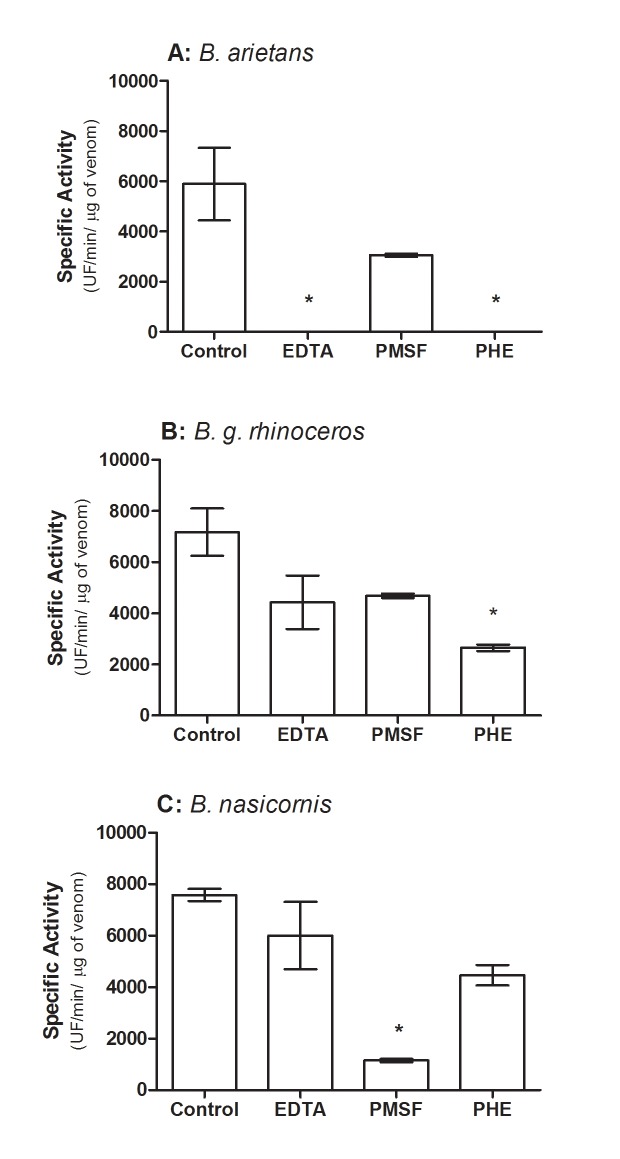
Proteolytic activity of *Bitis* ssp venoms on FRET substrate, the peptide Abz-RPPGFSPFRQ-EDDnp. The proteolytic activity of *Bitis* ssp venoms, pre-incubated or not with EDTA, PMSF or PHE was accessed by the hydrolysis of FRET substrate Abz-RPPGFSPFRQ-EDDnp (5 μM). All enzymatic assays were performed in quadruplicate and the results expressed as specific activity (UF/min/μg) ± SD. Statistical analyses were performed using one way ANOVA followed by the followed by Bonferroni multiple comparison test (*P< 0.05).

**Fig 5:**
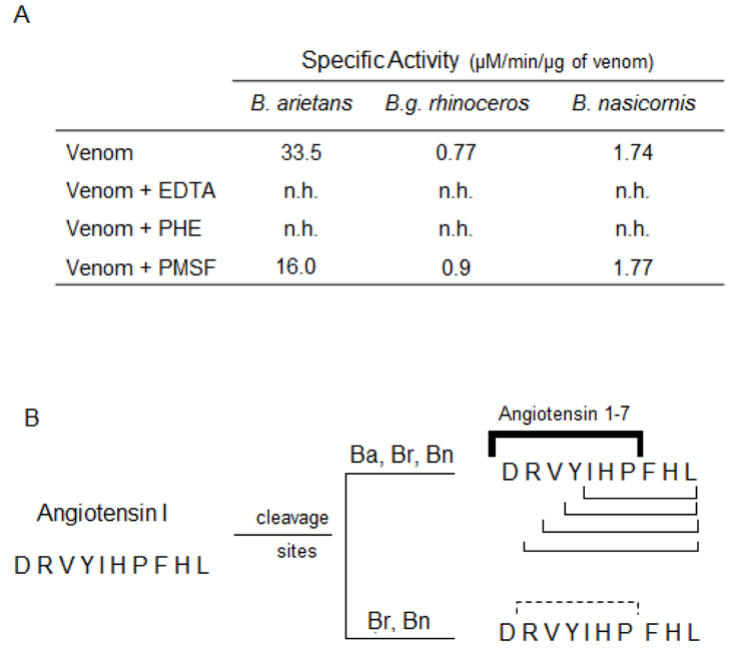
Cleavage of Angiotensin I by *Bitis* ssp venoms and identification of the cleavage sites on the primary sequence. **[A]** Angiotensin I (65 μM) was incubated at 37°C with 1 μg of *Bitis arietans* venom (1 h/37°C) or 5 μg of *B*. *nasicornis* or *B*. *g*. *rhinoceros* venoms (2 h/37°C) in phosphate buffer (50 mM sodium phosphate, 20 mM NaCl, pH 7.4). In parallel, the venoms were pre-incubated, 30 min prior the addition of angiotensin I, with PHE (5 mM), PMSF (5 mM) or EDTA (100 mM). **[B]** The hydrolysis products, collected during the reverse-phase chromatography, were submitted to mass spectrometry analysis. All venoms cleaved angiotensin I in different cleavage sites. The lines indicate the cleavage sites on angiotensin I primary sequence after treatment with the different *Bitis* venoms. The bold solid line indicates the cleavage point for angiotensin 1–7 generation and the solid lines indicate the other cleavage points observed after the treatment with the venoms. The dashed line indicates the sites on angiotensin I primary sequence cleaved only by *B*. *g*. *rhinoceros* and *B*. *nasicornis* venoms.

All *Bitis* venoms showed ability to cleave the FRET substrate, the peptide Abz-RPPGFSPFRQ-EDDnp (Fig. 4). *B*. *arietans* venom presented a specific activity of ~6000 UF/min/μg, which was completely inhibited by both EDTA and phenanthroline. *B*. *g rhinoceros* venom showed a specific proteolytic activity of ~7000 UF/min/μg, which was only inhibited by phenanthroline. *B*. *nasicornis* venom presented a specific proteolytic activity of ~7500 UF/min μg/, which was abolished by PMSF, but not for EDTA or phenanthroline. All the venoms also directly cleaved the angiotensin I generating angiotensin 1–7 (Fig. 5B). Besides angiotensin 1–7, the cleavage of angiotensin I generated several fragments with molecular weight varying from 770 to 1170 Da (Fig. 5B). *B*. *arietans* was more active in the cleavage of angiotensin I than *B*. *g*. *rhinoceros* and *B*. *nasicornis*. For all venoms, this proteolytic activity was completely abolished by PHE and EDTA, but not by PMSF (Fig. 5A).

### Neutralization of the enzymatic activities of *Bitis* venoms by the experimental antivenoms

In this study, it was also analyzed the potential of experimental horse antivenoms raised against *Bitis* venoms in neutralizing the different enzymatic activities described above. Two experimental horse antivenoms were tested, *i*.*e*., one against the venom of *B*. *arietans* (α-Ba antivenom) and other against *B*. *nasicornis* plus *B*. *rhinoceros* venoms (α-Br+ Bn antivenom). These two different antivenoms were produced considering the high number of accidents of *B*. *arietans* in in Africa and its venom strong immunogenecity, as compared with the *B*. *nasicornis* and *B*. *g*. *rhinoceros* snake venoms [21].

**Fig 6:**
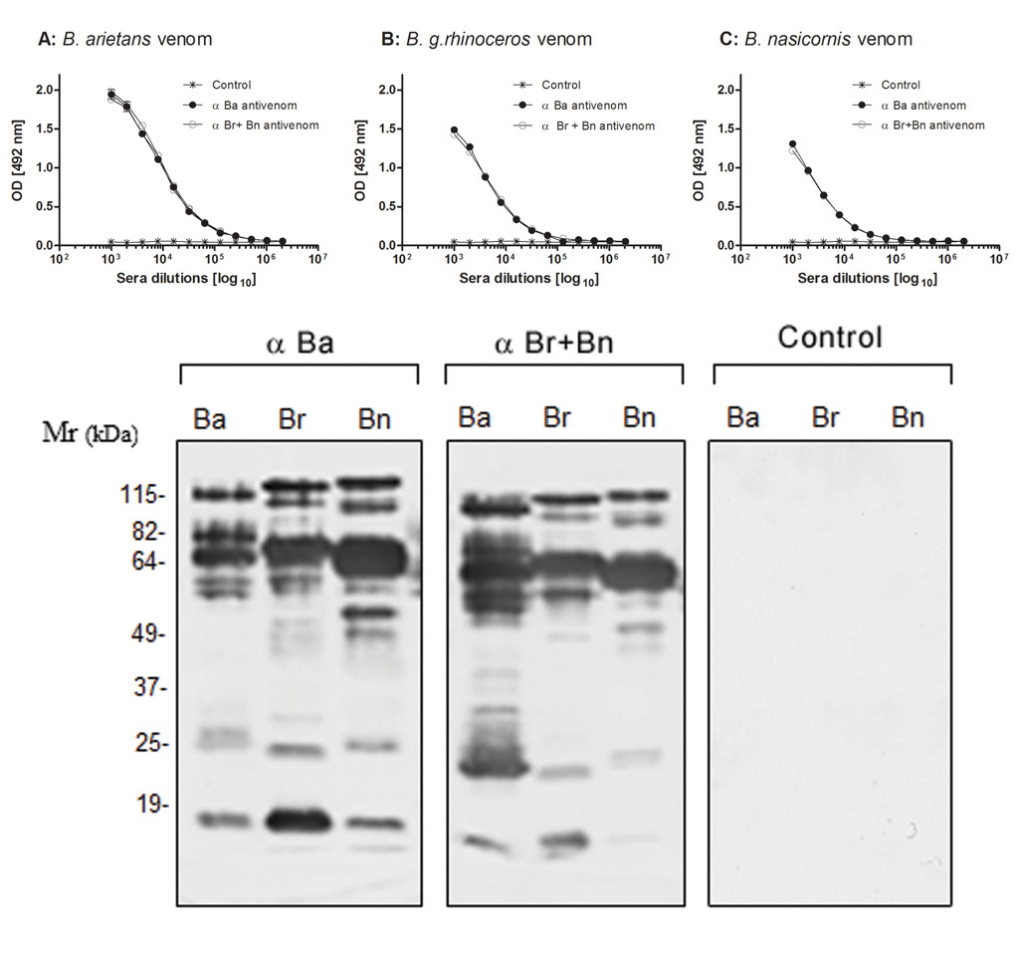
Titers and cross-reactivity of antivenoms raised against *B*. *arietans* and *Bitis g*. *rhinoceros* plus *B*. *nasicornis* venoms. **[A]** *ELISA*: ELISA plates were coated with 1.0 μg of the *Bitis* spp venoms/well and incubated with different dilutions of the horse experimental antivenoms produced against *B*. *arietans* venom (α-Ba antivenom), *B*. *g*. *rhinoceros* plus *B*. *nasicornis* venoms (α-Br + Bn antivenom) or with the botulinic toxin antiserum (control), followed by anti-horse IgG-HRPO-conjugated. The results were expressed as the mean of absorbance value ± SD. **[B]** *Western Blot*: venom samples (5 μg) from *B*. *arietans* (Ba), *B*. *g*. *rhinoceros* (Br) and *B*. *nasicornis* (Bn) snakes were separated by SDS-PAGE, electrotransfered to nitrocellulose membranes and incubated with the antivenoms raised against *B*. *arietans* (α-Ba), *B*. *g*. *rhinoceros* plus *B*. *nasicornis* (α-Br+Bn) or with botulinic toxin antiserum (control) diluted 1:2000 followed by GAH/Ig-AP. The reactions were revealed with NBT and BCIP.

α-Ba and α-Br + Bn antivenoms presented high specific antibody titers and cross-reactivity with others *Bitis* ssp venoms (Fig. 6A). Western Blot analysis showed that α-Ba and α-Br + Bn antivenoms efficiently immune reacted to a vast number of proteins in the crude venoms (Fig. 6B). Surprisingly, the western blot analysis showed that α-Ba antivenom better recognized the bands with low molecular weight in the venoms of *B*. *g*. *rhinoceros* and *B*. *nasicornis* than the α-Br + Bn antivenom (Fig. 6B).

The neutralizing effect of α-Ba and α-Br + Bn antivenoms on the enzymatic activity was tested by pre-incubating, 30 minutes at RT, the F(ab’)_2_ fragments, with each venom, prior the experiments. Neat F(ab’)_2_ fragments of α-Ba or α-Bn + Br sera incubated with the crude venoms were able to significantly reduced, both, the hyaluronidase and the PLA2 activities of the three *Bitis* spp venoms (Fig. 2).

**Fig 7:**
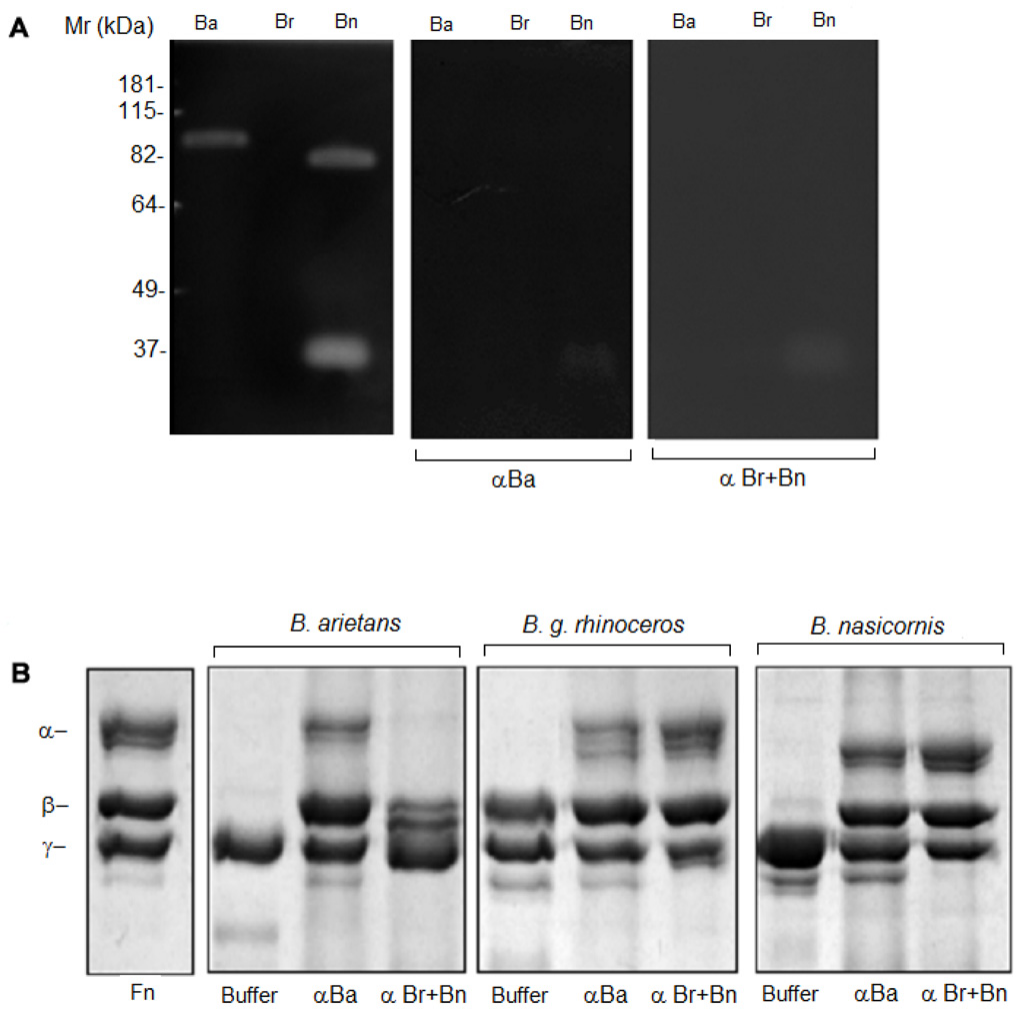
Inhibition of the proteolytic activity of *Bitis* ssp venoms by the experimental antivenoms. **[A]** *Bitis* spp venoms (20 μg) were pre-incubated (30’ at RT) with F(ab’)_2_ Ba (10 μL neat) or F(ab’)_2_ Br+ Bn (10 μL neat) antivenoms and ran under non-reducing conditions on a 10% polyacrylamide gel containing 1% gelatin type A. **[B]** One microgram of *B*. *arietans*, *B*. *g*. *rhinoceros* or *B*. *nasicornis* venoms were pre-incubated with 10 μL of neat sera prior incubation with 30 μg of human purified fibrinogen (Fn) then, ran under reducing condition in a 10% SDS-PAGE. The digestion of gelatin and the cleavage of fibrinogen were visualized by Coomassie Brilliant Blue staining.

**Fig 8:**
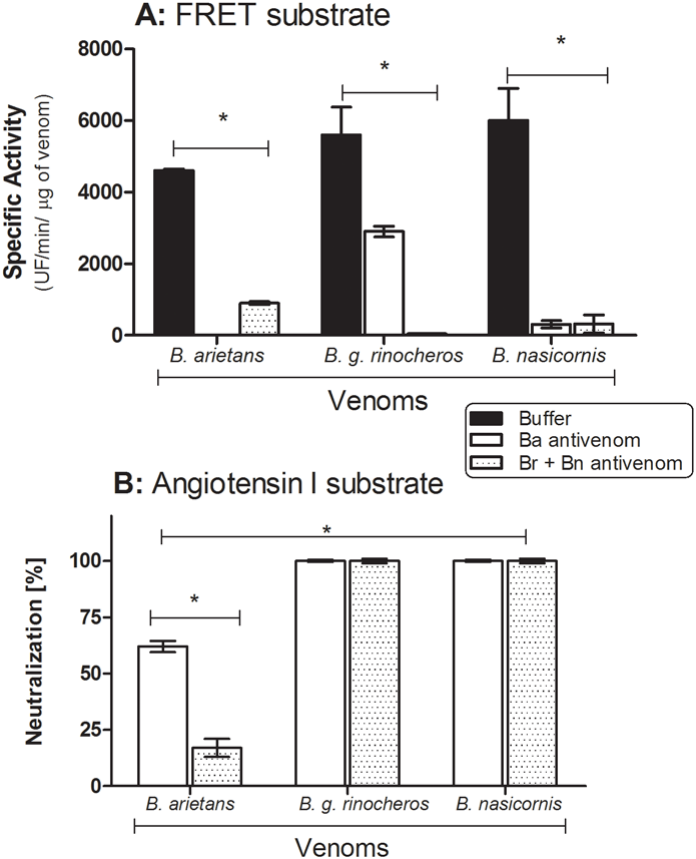
Inhibition of proteolytic activity of *Bitis* ssp venoms on FRET substrate, peptide Abz-RPPGFSPFRQ-EDDnp, and on Angiotensin I by antivenoms. **[A]** The proteolytic activity of *Bitis* ssp venoms, pre-incubated with 10 μL of neat antivenoms against *B*. *arietans* or *B*. *g*. *rhinoceros* plus *B*. *nasicornis* venoms, was accessed by the hydrolysis of FRET substrate Abz-RPPGFSPFRQ-EDDnp (5 μM/reaction). All enzymatic assays were performed in quadruplicate and the results expressed as specific activity (UF/min/μg of venom) ± SD. Statistical analyses were performed using two way Anova (*P< 0.05). **[B]** Inhibitory effect of the antivenoms α-Ba and α-Br + Bn (10 μL each) on the hydrolysis of Angiotensin I (65 μM) by venoms from *B*. *arietans*, *B*. *g*. *rhinoceros* and *B*. *nasicornis*. The experiments were made in triplicate. Statistical analyses were performed using two way Anova (*P< 0.05).

α-Ba and α-Br + Bn antivenoms could also abolish the gelatinolytic activity of *B*. *arietans* and *B*. *nasicornis* venoms; the low molecular weight band of *B*. *nasicornis* was also strongly reduced, when incubated v:v for 30 min prior the zymography (Fig. 7A). α-Ba antivenom (10 μL neat) prevented the cleavage of the fibrinogen by all the *Bitis* venoms (1 μg *per* reaction); however, the α-Br +Bn antivenom (10 μL neat) only prevented the cleavage of fibrinogen by *B*. *g*. *rhinoceros* and *B*. *nasicornis* venoms, but not by *B*. *arietans* (Fig. 7B). Both antisera (10 μL neat) were also able to inhibit the cleavage of the FRET substrate, Abz-RPPGFSPFRQ-EDDnp (Fig. 8A). The cleavage of angiotensin I by *B*. *g*. *rhinoceros* and *B*. *nasicornis* venoms was strongly abolished when both antivenoms were used. Nevertheless, the angiotensin I hydrolysis by *B*. *arietans* was weakly blocked by α-Br+Bn antivenom and partially inhibited by α-Ba (Fig. 8B).

## Discussion

Venoms from *Bitis* snakes remain poorly studied in spite of their involvement in an alarming number of life-threatening accidents in Africa. In the Sub-Saharan Africa most of the victims are rural workers engaged in traditional agricultural-pastoral labor, which live far from appropriate medical care, critically dependent on the availability of effective antivenom [26]. Furthermore, the antivenom therapy is not always reliable and effective to prevent the morbidity and mortality following the envenomation for all *Bitis* species. Even the most efficient and safe polyspecific antivenom, currently used for treating *Bitis* bite, the South African Institute of Medical Research (SAIMR) antivenom, is ineffective and should not be used in the treatment of bites caused by *B*. *atropos*, *B*. *cornuta* and *B*. *caudalis* while FavAfrique is mostly recommended for treatment of accidents by *B*. *arietans*, *B*. *gabonica* and *B*. *nasicornis* [26–28].

Specific therapies tackling the different pathways activated during the envenomation and not focused only on controlling the symptoms should be taken into account during the development of new treatments [2]. Nevertheless, the development of new treatments depends on in-depth understanding of the mechanisms by which the venom toxins cause pathology. The low efficacy of the current treatments for envenomation by all the species of *Bitis* is a result of the paucity of information available and the high diversity among the *Bitis* species. These facts lead us to investigate the biochemical and enzymatic characteristics of the three *Bitis* venom of medical importance, *Bitis arietans*, *Bitis gabonica rhinoceros* and *Bitis nasicornis* and focus on the toxic activities of these venoms that could be associated with the symptoms observed following the accidents with humans.

The electrophoretic profile showed that the protein components vary among the three *Bitis* species analyzed in this study, corroborating with data from previous biochemical [29], proteomic [5] and genomic [4] studies.

In many animal venoms, Phospholipase A_2_ (PLA_2_) is important for immobilization an digestion of the prey, as well as responsible for a variety of toxic and pharmacological actions, some of which are associated with the pathophysiology of snakebite envenoming (reviewed in [30]). All *Bitis* venoms here analyzed presented phospholipase activity, as also described by Calvete and colleagues in their proteomic study [4]. Hyaluronidase activity, which is involved in a large number of biological functions including the diffusion of venom toxins from the bite site into the tissues and circulation (reviewed in [31]), was also detected in the three Bitis venoms here studied. The venoms from *B*. *arietans* and *B*. *nasicornis* also contain enzymes with gelatinolytic activity, which are absent from *B*. *g*. *rhinoceros* venom. Similarly, it was reported that the venom of *B*. *parviocula* is also devoid of gelatinolytic activity [32]. It is interesting that the high molecular weight band with gelatinolytic activity, in both venoms, was inhibited by PHE and PMSF suggesting that these components are serineproteinases with some metal dependence to maintain their structural integrity or catalytic site’s function or, that these venoms present serine- and metalloproteinases with similar molecular mass. However, the band with lower molecular weight and gelatinolytic activity, detected in *Bitis nasicornis* venom, was inhibited by PMSF but neither affected by EDTA nor PHE, indicating that a serineproteinase is responsible for this activity. Some toxins isolated from venoms can have this dual effect upon certain substrates and be inhibited by both PMSF and PHE [33] while others, as the calcium-and zinc-independent gelatinases can be unaffected by EDTA, PHE nor PMSF [34]. The metalloproteases present in *B*. *arietans* and *B*. *g*. *rhinoceros* venoms also displayed hydrolytic activity on peptide Abz-RPPGFSPFRQ-EDDnp whilst, in the venom of *B*. *nasicornis*, this cleavage was mediated by serineproteinases. Interestingly, the results obtained with *B*. *nasicornis* are comparable with previous report using the venom of *Bothrops jararaca* [35].

Some studies showed that venoms from *Bitis* ssp interfere with the coagulation cascade by direct cleavage of its proteins or inducing its over-activation [36–38]. Our data shows that metalloproteinases present in the venoms of *B*. *arietans* and *B*. *nasicornis* cleave the α- and β- chains of the fibrinogen whilst, the metalloproteinases of *B*. *g*. *rhinoceros* venom cleave only the fibrinogen α-chain, as thrombin. Metalloproteinases with fibrinogenolytic activity are present in a large number of snake venoms [39], but unlike the thrombin, these enzymes are not inhibited by serpins, the natural inhibitor of fibrinogenolytic serineproteinases [40]. In normal conditions, the cleavage of fibrinogen by thrombin results in clot formation that facilitate platelet aggregation and/or the formation of monomers of fibrin [41]. Cleavage of fibrinogen’s α- and β- chains was also observed by Sanchez and colleagues [32] who demonstrated that *B*. *arietans* and *B*. *parviocula* venoms are very hemorrhagic and interfere with the coagulation cascade by delaying the activated clotting time and clotting rate (time in which fibrin formation begins). In addition, *Bitis nasicornis* venom also has an anticoagulant effect *in vitro* as demonstrated by abnormalities in the pro-thrombin time and prothrombin consumption [36].

Low blood pressure is also commonly observed in patients envenomed by *Bitis* ssp venoms [42, 43], therefore, our interest in investigating the presence of vasopeptidases, enzymes responsible for the generation or inactivation of vasoactive peptides. Metalloproteinases from *Bitis arietans* venom cleaved angiotensin I (DRVYIHPFHL) with high specificity generating, preferentially, angiotensin 1–7 (DRVYIHP) whilst, *B*. *g*. *rhinoceros* and *B*. *nasicornis* venoms generated angiotensin 1–7 with lower specificity. Angiotensin 1–7 is a counter-regulator of cardiovascular effects of angiotensin II that contributes to vasodilatation [44], increase of nitric oxide and acts upon the platelets causing inhibition of adhesion and aggregation [45]. In addition, all *Bitis* venoms also generated other fragments such as RVYIHPFHL, VYIHPFHL and YIHPFHL, most of them bioactive [46], suggesting the presence of a metal-dependent protease with similarity to aminopeptidases. These data corroborate with prior findings of Vaiyapuri and colleagues [47] that purified and characterized an aminopeptidase from *Bitis gabonica rhinoceros* venom, named rhiminopeptidase, that removes basic and neutral aminoacids from the N-terminus of the peptides. Moreover, *B*. *g*. *rhinoceros* and *B*. *nasicornis* generated another fragment RVYIHP (see Fig. 6B).

The proteolytic activity of *Bitis* venoms described in this study was efficiently inhibited by the experimental antivenoms raised against *B*. *arietans* and *B*. *g*. *rhinoceros* plus *B*. *nasicornis* venoms. The only exception was the gelatinolytic activity of the serineproteinase with ~30kDa present in *B*. *nasicornis* venom. In all the experiments, α-Ba and α-Br + Bn sera inhibited the proteases activity of the venom against it was produced but also the activity of the venom from others species of *Bitis*, except for the fibrinogenolytic activity of *B*. *arietans* venom that was not inhibited by α-Br + Bn. The inhibition by the antivenoms was specific, since horse F(ab’)_2_ fragments produced against a non-related toxin, the botulinic toxin, did not abolish any of the enzymatic activities reported here for the *Bitis* venoms.

Both antivenoms were also able to strongly inhibit the PLA_2_ activity from *B*. *arietans* and *B*. *g*. *rhinoceros* venoms. However, the phospholipase activity of *B*. *nasicornis* venom was only weakly blocked by the two antivenoms. In 2010, Calvete and colleagues [48], using immunodepletion approach, showed that the majority of the proteins in the crude venom from *B*. *arietans*, *B*. *gabonica*, *B*. *rhinoceros* including metalloproteinase, serinoproteinase, C-type lectin among others immunoreacted with the immunoglobulins of the Echi-Tab-Plus-ICP antivenom. Interestingly, they also demonstrated that this antivenom could only partially react with some phospholipases A_2_ and disintegrins.

Envenomed patients seldom recognize the species of snake involved in the accident and this identification is normally based on the symptoms that are common among the species of the same genus. Overall, our data suggest that an efficient venom neutralization of the three species of medical importance in Africa could be achieved by pooling the two horse experimental antivenoms, *i*.*e*., the one against *B*. *arietans* and the other against *B*. *gabonica rhinoceros* plus *B*. *nasicornis* venoms.

In conclusion, in this report we have functionally characterized the venom from three species of *Bitis* involved in accidents with humans in the Sub-Saharan Africa. These venoms possess a combination of proteases that direct affect the coagulation system via cleavage of fibrinogen, which can, consequently, prevent platelets aggregation and the systems involved in the modulation of blood pressure regulation and salt balance via generation of active vasopeptides. We also demonstrated that some of the deleterious activities present in *Bitis* venoms can be efficiently blocked by antivenoms produced against *B*. *arietans* or *B*. *g*. *rhinoceros* plus *B*. *nasicornis* venoms.
